# Discovering Subgroups of Patients from DNA Copy Number Data Using NMF on Compacted Matrices

**DOI:** 10.1371/journal.pone.0079720

**Published:** 2013-11-20

**Authors:** Cassio P. de Campos, Paola M. V. Rancoita, Ivo Kwee, Emanuele Zucca, Marco Zaffalon, Francesco Bertoni

**Affiliations:** 1 Dalle Molle Institute for Artificial Intelligence (IDSIA), Manno, Switzerland; 2 Lymphoma and Genomics Research Program, Institute of Oncology Research (IOR), Bellinzona, Switzerland; 3 University Centre of Statistics for Biomedical Sciences (CUSSB), Vita-Salute San Raffaele University, Milan, Italy; 4 Swiss Institute of Bioinformatics (SIB), Lausanne, Switzerland; 5 Lymphoma Unit, Oncology Institute of Southern Switzerland (IOSI), Bellinzona, Switzerland; Memorial Sloan Kettering Cancer Center, United States of America

## Abstract

In the study of complex genetic diseases, the identification of subgroups of patients sharing similar genetic characteristics represents a challenging task, for example, to improve treatment decision. One type of genetic lesion, frequently investigated in such disorders, is the change of the DNA copy number (CN) at specific genomic traits. Non-negative Matrix Factorization (NMF) is a standard technique to reduce the dimensionality of a data set and to cluster data samples, while keeping its most relevant information in meaningful components. Thus, it can be used to discover subgroups of patients from CN profiles. It is however computationally impractical for very high dimensional data, such as CN microarray data. Deciding the most suitable number of subgroups is also a challenging problem. The aim of this work is to derive a procedure to compact high dimensional data, in order to improve NMF applicability without compromising the quality of the clustering. This is particularly important for analyzing high-resolution microarray data. Many commonly used quality measures, as well as our own measures, are employed to decide the number of subgroups and to assess the quality of the results. Our measures are based on the idea of identifying robust subgroups, inspired by biologically/clinically relevance instead of simply aiming at well-separated clusters. We evaluate our procedure using four real independent data sets. In these data sets, our method was able to find accurate subgroups with individual molecular and clinical features and outperformed the standard NMF in terms of accuracy in the factorization fitness function. Hence, it can be useful for the discovery of subgroups of patients with similar CN profiles in the study of heterogeneous diseases.

## Introduction

Discovery of disease subtypes or of subgroups of patients sharing common characteristics is a challenging task in biomedical research, especially in the study of complex and heterogeneous genetic disorders. The purpose of these analyses is, for example, to allow for a better prediction of the survival time and treatment decision, to understand the reasons for drug resistance, or to provide general insights about the biological mechanisms of the disease for finding diagnostic biomarkers or drug targets that are specific to a subgroup.

One type of genetic aberration is the change in the DNA copy number (CN). In human beings, the normal CN is two for the majority of the genome since, for each chromosome, one inherits a copy from each of their parents. Thus, CN changes are defined as lesions in which the number of copies is different from two. We can classify these CN aberrations into the following four categories: homozygous deletion (loss of two copies), heterozygous loss (loss of one copy), gain (number of copies equal to three or four), amplification (number of copies greater than four), see Table 1. The identification of these types of lesions is important, for example, in cancer studies. In fact, the gain or the amplification of an oncogene would determine the overexpression of the corresponding protein and thus, for example, to uncontrolled cell growth. The loss of a tumor suppressor gene can make, for instance, the cells resistant to apoptosis. Another type of genetic lesion can be identified through the values assumed by single-nucleotide polymorphisms (SNPs). A SNP is a variation of the DNA sequence at a single base-pair location (there is a pair of nucleotides at each genomic locus, since normal CN is two). The two paired nucleotides can assume only two possible alleles (for simplicity, we call them A and B) among the four basis. If they assume the same allele, then the value of the SNP is AA or BB and the SNP is called homozygous, otherwise its value is AB and the SNP is called heterozygous. The loss of heterozygosity (LOH) lesion can be displayed as a long stretch of homozygous SNPs. SNP microarrays are able to measure both the CN and the LOH at hundred thousands or even millions of SNPs along the genome [1].

**Table 1:** Summary of copy number lesions that are considered.

CN	name of the lesion
0	homozygous deletion
1	heterozygous loss
2	normal copy number (no lesion)
3 or 4	gain
>4	amplification

This table shows the definition of the four types of copy number (CN) aberrations that are considered in this work.

In order to identify subgroups of patients with common patterns of CN and LOH lesions, non-negative matrix factorization (NMF) is a well-known approach [2], [3]. It has been widely used in a variety of areas and problems, including many in Computational Biology (we refer to [3] for a broad view of its applications). While NMF has been mostly applied to continuous data such as gene expression, it can also be used with discrete data such as CN lesions by optimizing an appropriate divergence function [4]. NMF is particularly suitable for high-dimensional CN and LOH data because of sparseness and repetitiveness in the matrices representing these lesions [3], [5]–[9]. Because of that, it is able to produce clustering results where the most important characteristics of the data set are emphasized. However, as the dimension of data increases with new microarray technology, the harder (in both time and accuracy) becomes to run NMF over the full data matrix (we refer to the matrix rows as patients/samples and to the matrix columns as their CN information at different physical genomic locations). Current computers can perform one run of NMF in about one day (note that we need to perform hundreds of runs) with matrices in the order of 10^4^ columns, which represents at least two orders of magnitude less than what current microarray technology can output. The most common approach to address this problem is to restrict the attention to a smaller number of pre-selected columns, and perform the NMF analysis only with such columns. These columns are either selected in some ad-hoc manner (for example, one every few columns, usually because of their physical proximity), or by applying some procedure which identifies groups of similar columns (using statistical tests or other methods with the same aim, such as minimum common regions, principal component analysis, etc) and keep only one column for each group. Such ideas make it possible to apply the factorization to such large matrices of data, otherwise computationally unfeasible. We analyze this task and propose a way of “compacting” the columns such that the additional error introduced by such a compaction is minimized. Our so called *Compact-NMF* differs from previous uses in an essential way: we aim at factorizing the data matrix in a way that the divergence to the whole data matrix is minimized, while a naive approach, not being able to run NMF over the whole data matrix, would work on a submatrix of it, and thus would produce a factorization that minimizes the divergence to that submatrix (we call this idea *Standard-NMF*). Using real data, we show that Compact-NMF produces results that are in line with running NMF over the whole data matrix, while Standard-NMF has a considerable degraded accuracy, which we will define as the fitness of the factorization later on. The implementation of Compact-NMF is available at http://www.idsia.ch/~cassio/compactnmf/.

Using these techniques, we analyze data sets of patients affected by diffuse large B-cell lymphoma (DLBCL), by breast cancer with the *HER2* gene amplification, and by medulloblastoma. The first data set consists of SNP microarray data (Affymetrix GeneChip Human Mapping 250K NspI) from 166 DLBCL samples [10]. Since genomic aberrations can be shared by different lymphoma subtypes, we enlarge the data set by adding data from patients affected by other types of B-cell leukemia and lymphomas [11]. The use of a large data set of 533 samples with similar diseases (which increases the number of rows in the matrix) may improve the clustering, similarly to what was shown for the definition of recurrently aberrated regions in [11]. The second data set regards aCGH data (NimbleGen Human CGH 385K Whole-Genome Tiling v1.0 Array) from 201 DLBCL patients of [12]. The third data set consists of 200 breast cancer patients' data [13] obtained with BAC microarrays (produced by the SCIBLU Genomics Center), in three resolution formats (32K, 33K, and 38K). Finally, the fourth data set is a huge collection of very high-throughput microarray CN profiles. In fact, it comprises the data of 1087 medulloblastoma patients [14] obtained with the Affymetrix Genome-Wide Human SNP Array 6.0 platform (about 1.8 million probes). With the aim of evaluating the quality of the separation, we show that the subgroups identified by our clustering procedure are associated with other known clinical information of the patients (not disclosed to the clustering procedure itself), such as molecular subtypes of the corresponding diseases. As with most unsupervised clustering techniques, the choice of the *correct* number of subgroups is a problem by itself and has to be analyzed with attention. We employ a wide selection of well-established quality measures and discuss some new criteria. All of them are unsupervised, which reduces the chance of overfitting.

## Methods

Non-negative Matrix Factorization [2] is a technique to reduce the dimensionality of a data set, and can be justified as a clustering algorithm given its non-negative characteristic (Principal Component Analysis, for instance, is much faster but does not have the same properties because it allows negative values in its decomposed matrices and makes the result non-interpretable for clustering). The goal is to produce a pair of matrices of smaller rank than the original one which multiplied produce a good approximation of the latter. Let 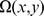 denote the space of matrices with dimension 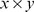, 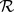 the real numbers and 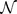 the natural numbers. A matrix 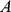 of 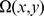 can be seen as a function 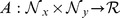, with 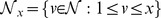. Thus, an element in the position 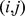 of 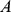 is simply denoted by 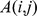. Let 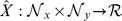 be defined as follows: 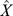 = *W⋅H*, that is,
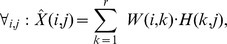
where 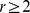 is a given integer with the desired rank of the factorization, 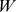 and 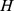 are decomposed matrices with dimensions 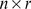 and 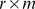, respectively. The standard cost/divergence function used within NMF when it is seen as a maximum likelihood estimation and data are assumed to be produced by a Poisson distribution (which is our case, given that we consider the estimated number of DNA copies at each SNP probe) is:
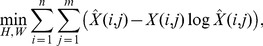
(1)subject to 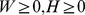, where 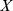 is the given 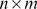 input matrix and 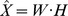 is the estimated matrix. This divergence function measures the quality of 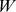 and 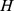 as a decomposition of 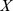, and can be interpreted as the Kullback-Leibler (KL) divergence between distributions represented in the matrices 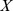 and 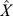 (if one normalizes these matrices). Hence, the accuracy of NMF can be defined as the result of Equation (1) that is achieved by the factorization.

The problem of optimizing the divergence function (that is, solving NMF) is known to be a computationally hard task (in computational complexity terms, it is known to be NP-hard for 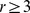 [15]). The function is non-convex and there are multiple local optima. In fact, under the KL divergence, NMF has been shown to be a similar task as the Expectation–Maximization technique for learning parameters of multinomial distributions with latent variables [16]. This justifies the development and use of efficient local optimization techniques, as well as multiple-start methods to avoid being trapped in a local minimum of the divergence function. The starting point for the optimization is the initial solution that one can choose to fill up the matrices 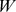 and 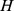 with values. Moreover, it is known that the problem is not identifiable, that is, there might be multiple global optimal solutions [17]. Hence, multiple-start methods can not only avoid local minima, but should be integrated somehow to produce a better overall solution for the optimization problem, as finding a single global minimum solution is not the best one can do [18]. Essentially, when one takes the most accurate run of NMF as the final result, they would be relying on a single solution, even if there might be many distinct solutions which achieve equal or only barely worse accuracy than the best one, and yet these solutions might be composed of quite different matrices 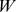 and 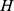. This issue can be explained by the aforementioned relation between NMF and learning parameters of multinomial distributions with latent variables [16].

### NMF as a Clustering Method

NMF can be used for clustering by considering each row 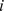, 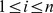, of the matrix 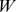 as an element to be clustered, and the row itself as weights by which element 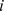 should be assigned to each cluster 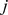, 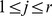 (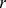 is the number of clusters/groups). The simplest idea is to assign each element 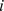 to the cluster 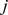 with largest weight, that is, the clustering 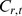 for the 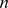 elements (rows) of the input is defined as 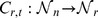 such that 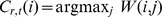, where 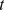 is just an index to identify results from distinct NMF runs.

Using NMF for clustering leads to the *permutation* problem, if one wants to put together the results of many runs of NMF from multiple distinct starting points. This problem regards the fact that different runs of NMF can lead to distinct assignments of clusters that differ only by a permutation of the columns of 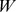 (respectively rows of 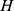), while achieving the same value of the divergence function. Moreover, it is not trivial to combine different runs of NMF, for example, by combining the corresponding positions of the matrices 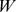 and 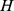 of the runs, as each run might regard a distinct permutation of the matrices. In order to address the permutation problem, we follow the standard post-NMF approach of combining clustering results by building up an overall similarity matrix, which contains the number of times the elements were clustered in the same cluster. As this approach performs calculations that are separate for each run of a multiple-start NMF, the result is coherent. More formally, let 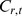, for 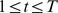, denote the clustering result of the NMF procedure in run 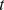. The similarity matrix 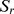 with dimension 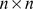 is defined as
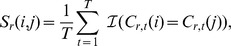
where 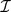 is the indicator function. Now, the similarity matrix 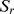 can be used as input of another clustering technique, such as hierarchical clustering [19] or partition around medoids [20], in order to produce a final clustering result. Note that any clustering technique that is able to produce a clustering out of a similarity matrix might be employed here. In fact, there is no significant difference between the methods we just mentioned, since the matrix 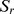 produced by many runs of NMF is already a robust measurement of the similarity of the elements to be clustered, and thus the effect of using distinct clustering methods over 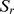 is greatly reduced. We denote by 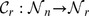 the final result of the clustering over 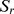, associating each row of the initial matrix to a cluster number between 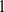 and 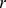. In this work, we employ hierarchical clustering for this purpose.

Another important issue with NMF is its scalability to large matrices. Obviously this depends intrinsically on the method to optimize the divergence function. The most traditional method to optimize it is the iterative procedure of [4] (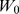 and 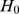 are given as the starting point to the algorithm):
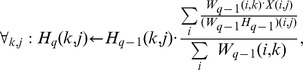


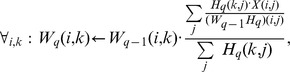
for 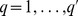 until convergence (or until a maximum number of steps is reached). Finally, 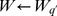 and 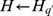. This method is proved to converge to a stationary point (usually a local optimum) of the minimization problem. Note that the most expensive operation executed by the algorithm while updating 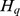 is the multiplication of matrices 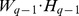 (respectively 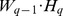 in the computation of 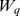). Even with an optimized implementation, each step takes approximately cubic time in the matrix dimensions. We provide an optimized C++ implementation of this algorithm, which also works as a library package for the R language (see http://www.idsia.ch/∼cassio/compactnmf/). The implementation is fast and can be directly invoked as a stand-alone application, which is desirable when running it on a grid or parallel computer environment (given the need of running the optimization several times with distinct starting points to obtain a robust similarity matrix).

### Compact-NMF

Even if we use a fast implementation and a computer grid to parallelize the runs of the algorithm, the maximum matrix dimension that current computers can handle is quite limited, because of the intrinsic complexity of the problem. Hence, we propose to reduce the dimension of the matrix 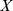 by compacting the information of some of its columns (that is, SNP probes in the case of dealing with CN data) even before applying NMF. The idea is that we are given computational resources that are able to solve an NMF instance up to matrices 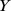 of dimension 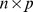, but we still want to approximate the (eventually much larger) 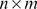 matrix 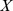. This situation occurs often, since current computers have severe limitations in both memory and time when the dimension is too large. For instance, a few thousands of columns are feasible for running NMF, but hundreds of thousands are not. In such cases, the most widely used preprocessing ideas that are applied before running NMF discard part of the columns, using either a systematic method (for example, choosing one every 50 probes, or performing feature selection, etc) or a statistical approach until the number of remaining columns becomes computationally solvable. However, when one applies NMF after any procedure that discards columns without a proper procedure to keep some guarantees, they are in fact approximating the reduced matrix in a non-optimal way, instead of approximating the original larger matrix. We propose here a formulation called *Compact-NMF* that directly approximates the original matrix 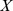, still by solving the smaller NMF over 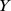. A detailed explanation of the reasoning behind the method is given in the Supporting File S1. The Compact-NMF method solves the problem:
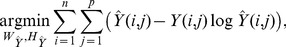
(2)where 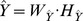 is being optimized, 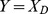, and
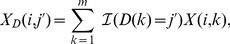
(3)with 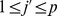 and 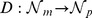 being a partition of the 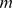 columns of 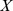 into 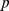 bins. In words, it means that we sum columns that are put together in the same bin 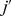, defined by the partition 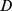. Expression (3) is justified in more details in the Supporting File S1. We define the partition 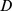 using the hamming distance between columns, that is, 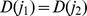 whenever 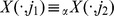, with the operation 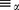 (for a given non-negative integer 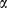) defined as the condition that the Hamming distance between the columns is not superior to 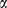:



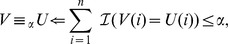
with 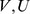 generic columns (each containing 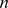 values). Then we choose 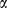 that yields matrices of reasonable size according to our computational resources. When dealing with categorical or discrete features, for example, the presence or the absence of an aberration, or the number of copies of DNA (which can only assume a few distinct numbers), hamming distance is a natural choice to measure the similarity between columns. Finally, even 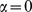 would (possibly) produce great speed up, as equal columns would be merged together, and the NMF over 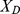 would solve the very same problem as the original NMF over 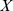 (see the Supporting File S1 for details). We call this particular version *Full-NMF*, as it minimizes the divergence of the whole matrix, but still runs NMF over the smallest possible lossless matrix. In our experiments with real data sets, we perform a slightly more aggressive reduction than lossless by allowing columns to differ in at most 1% from each other in order to consider them equivalent. The choice of 1% was taken empirically in order to obtain matrices of about 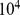 columns, which is the size that computation could be performed in a reasonable amount of time (about one day for one NMF run over all ranks). In other words, we compact the original matrix into a new (much smaller) matrix that represents almost the same information. In the worst case, this corresponds to losing the information of about 1% of the original data. Some loss of data is inevitable if it is to run the method in feasible time, as this is a very demanding computational task. Yet, this procedure loses less information than other ideas mentioned in the beginning of this section. In order to select which columns to put together, we use a straightforward greedy algorithm that goes through the columns and looks for those that have a small Hamming distance among each other, but any other idea would equally suffice. Mainly, the benefit of the proposed compaction is that it is optimal towards minimizing the divergence function, so theoretically preferred to other preprocessing ideas that reduce the matrix dimension before running NMF. Its main limitation regards the amount of similarity between columns of the original matrix: if all columns are very different from each other, then the only way to compact the matrix is to combine columns that are not necessarily similar, which might decrease accuracy. This is not a particular issue of this compaction but of any preprocessing idea to reduce the matrix dimension. We empirically demonstrate the accuracy of such ideas later on by comparing the different versions of NMF.

### Choosing the Number of Clusters

An important problem in clustering is the choice of the number of clusters 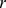 (also called rank in the case of NMF). Many criteria have been proposed in the literature, such as the cophenetic correlation coefficient (if one is using hierarchical clustering to put NMF runs together) [19], the elbow of the Mean Squared Error (MSE) and of the Area Under the Curve (AUC) [21], the Davies-Bouldin index [22], the Gamma statistic [23], the Hubert-Levin test [24], the intra-cluster scatter [25], Silhouette plots and averages [26], among others. These criteria do not necessarily agree with each other. One might simply pick the criterion of their preference, but this is an arbitrary choice. Even if the criteria agree, they are not designed to discriminate a cluster that might be strongly different from the others in a given clustering of rank 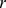, but only to score the separation quality of the rank as a whole (that is, all clusters against each other). In other words, if there is a single cluster that considerably differs from the others, while the remaining clusters are not well-separated, these criteria will probably fail to identify it. Nevertheless, identifying a single well-separated cluster within a rank is in general an important task, sometimes more important than choosing the best rank itself. Fortunately, most of these criteria are decomposable, that is, they define an overall quality measure of a clustering result based on the quality of each of its clusters, so they can be adapted to account for such strong clusters. We propose the following criterion, which is based on an *intra-cluster similarity* [26]:
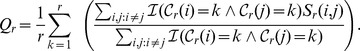



In words, for each cluster 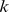 in the rank 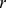, the average similarity between the elements of cluster 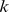 is computed (this is the internal ratio in the formula), and finally we take the mean of these averaged similarities. When using this criterion with NMF, the value 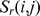 from the similarity matrices can be seen as the probability of rows 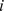 and 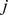 to be grouped together, so the average value within each cluster corresponds to the mean probability of two elements (patients in our case) to be together. This measure is permutation invariant. Moreover, 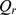 can easily be adapted to use, instead of the mean, the median or the maximum values over the clusters. For the purpose of identifying robust subgroups (even when the overall rank is not robustly separated), we are especially interested in the maximum value obtained by a subgroup, as this particular subgroup might represent some important characteristics of the population. We refer to this idea as *maximum intra-cluster similarity*. It is a relevant criterion when one wants to compare the performance of a given subgroup (in a given rank) against the others (of the same rank). However, we emphasize that it does not consider the inter-cluster similarity in its formula, thus it tends to value subgroups that have very similar samples, regardless of other samples (not in the same subgroup) that might be also similar to them. All in all, we do not intend to propose 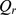 as a solution to the selection of the number of clusters, but as another relevant measure that may be suitable depending on what the analysis is aiming at. So far, we believe that the most general way to address the problem is to consider many measures that evaluate different aspects (in the sense of what they measure) and choose the number of clusters that best agrees with them altogether, including the just defined intra-cluster similarities.

### Assessing the Quality of the Factorization

In order to assess the quality of the factorization and the overall results, we perform two verifications: (i) the number of NMF runs with random initial guesses is checked to show that the fitting has reached a stable good solution; (ii) the subgroups generated by NMF are analyzed against two distinct features, namely disease subtypes and survival outcome of patients. As described before, many NMF runs are used to build the similarity matrix. Obviously, the more runs the more precise is the similarity matrix, because each run is expected to produce a good factorization of the initial matrix. Given a certain number 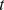 of runs and their outputs, one might check the amount of change in the similarity matrix by running NMF once more, that is, increasing 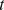 by one. To assess the need of additional runs, we take subsets of the 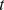 runs and compute the mean squared error between their similarity matrices and the one created by 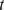 runs. If the difference is small, then the similarity matrix probably will not change considerably if more NMF runs were included, meaning that we have already reached a sufficient number of runs.

With the aim of checking the meaningfulness of the clustering produced by NMF from genomic data, we verify whether it correlates with some clinical or biological characteristics. When these characteristics are categorical, such as disease types and subtypes, we perform a Fisher exact test on the contingency table. When correlating right-censored survival outcome, we estimate survival curves using the Kaplan-Meier estimator and perform log-rank test and Peto & Peto's generalization of the Wilcoxon test to assess the differences among curves. Because no clinical information is used during the factorization (NMF is applied as an unsupervised method), there is no overfitting regarding this information, and the tests show how strong is the association between the NMF clustering of genomic data and the biological/clinical data.

## Results and Discussion

We show the performance of our procedure in the analysis of two data sets of patients with diffuse large B-cell lymphoma (DLBCL), one with breast cancer and one with medulloblastoma. In the first data set (called Data set 1), the data of 166 DLBCL cases[10] were analyzed together with additional 367 B-cell neoplasia in order to achieve a more reliable clustering [11]. For each sample, the CN and LOH data (approximately 250,000 probes, each interrogating a single SNP) were obtained with Affymetrix GeneChip Human Mapping 250K NspI array, and then preprocessed with CNAT 4.01 [11]. After that, the CN profiles have been estimated with the modified Bayesian Piecewise Regression (mBPCR) method [27]. Finally, the CN aberrations were divided into two major categories corresponding to a decrease or increase of number of copies, that is, CN 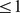 and CN 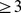. As a consequence, for each probe, the CN information was separated into two different features (defined as columns in the NMF formulation), each corresponding to one of the two major categories. In the features representing CN 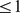 (respectively CN 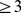), we encoded the aberrations in the following way: normal is 0, heterozygous loss (respectively gain) is 1, homozygous deletion (respectively amplification) is 2. The choice of these numbers was motivated by their clear counting interpretation in terms of severity of aberrations, which fits with the use of the NMF under the assumption that data are generated from a Poisson distribution. Moreover, the separation of the aberrations into two features was necessary because the Poisson distribution is not symmetric, so keeping all of them together in one feature (with the natural encoding from 0 (homozygous deletion) to 4 (amplification)), would imply a preference towards lesions of the type CN 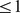, which we obviously do not want. The LOH profiles were estimated with dChip [28] and copy-neutral LOH lesions (that is, regions with LOH but normal CN) were denoted by 0.5 in the feature representing CN 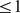, due to the biological effect of this type of lesion.

Regarding the 201 DLBCL patients of [12] (named Data set 2), we obtained the aCGH data from the Progenetix database ([29]; www.progenetix.org). The data were already preprocessed, segmented and discretized in normal CN, increased CN (CN 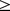 3) and decreased CN (CN 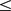 1). Furthermore, the profiles of 200 *HER2*-amplified (*HER2+*) breast cancer patients [13] (Data set 3) were obtained from the ArrayExpress database (E-GEOD-21259, www.ebi.ac.uk/arrayexpress). The data were only already preprocessed, thus, we segmented and discretized them in normal, increased and decreased CN, by following the methods described in [13]. Finally, the copy number SNP microarray data of 1087 patients affected by medulloblastoma [14] were obtained again from the Progenetix database. The data were already preprocessed, segmented and discretized as for Data set 2. In all of these cases (Data sets 2, 3 and 4), and similarly to the analysis of Data set 1, for each probe, we separated the CN data into two features corresponding to the two major categories of aberrations (CN 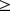 3 and CN 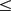 1). For each feature, we encoded the normal state as 0 and the presence of the aberration as 1 (in this case, we did not have information to distinguish between gain and amplification, and between loss and homozygous deletion).

### Compact-NMF versus Standard-NMF

**Figure 1:**
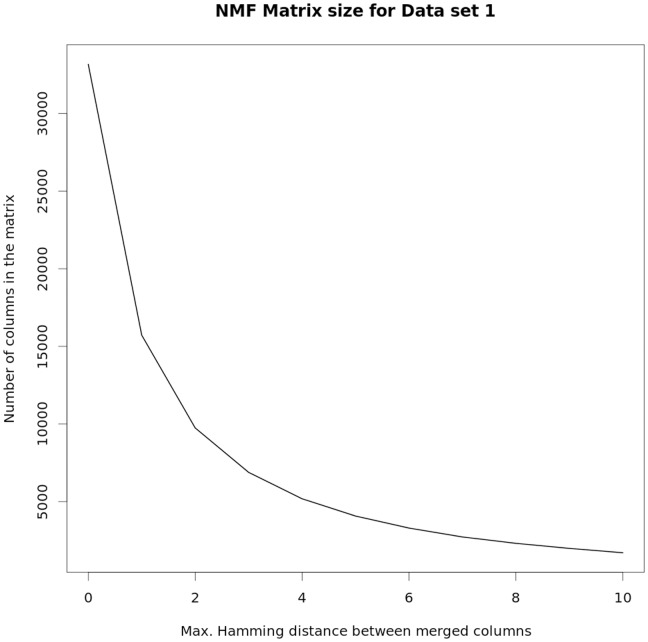
Size of matrices to run NMF according to the Hamming distance to consider equivalent columns. The matrix produced by using the DLBCL samples of Data set 1 as rows and their DNA copy number profiles as columns was subjected to the compaction procedure, according to the similarity between columns based on Hamming distance. As higher the maximum allowed Hamming distance used to merge columns as lower the number of resulting columns. Later, matrices of those dimensions are used as input for the NMF.

In order to assess the relevance of Compact-NMF, we have analyzed the divergence value (from Equation (1)) that Compact-NMF and Standard-NMF achieved in comparison to Full-NMF (the lossless NMF procedure representing the accuracy obtained by running NMF without discarding any information). We have worked with Data Set 1 for this purpose, as it is a large data set (both in number of patients and probes) but still manageable in order to perform many rounds of tests. Figure 1 shows the number of columns (probes) that are obtained from Data set 1 when applying the merging procedure for Compact-NMF, using the similarity function with Hamming distances as defined in the Section Methods. Notice that, for a fair comparison, the number of remaining columns is the same for both Compact-NMF and Standard-NMF (so Figure 1 shows a single curve), because we employ the same similarity function to both cases in order to decide the merging (or discarding in the case of Standard-NMF), even though Compact-NMF and Standard-NMF handle differently how to treat them.

From a total of more than 500 thousand columns (twice 250 thousand probes because CN data have been separated into two features as described previously), a lossless compaction (that is, using 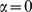 with Compact-NMF) generated a matrix with around 33 thousand columns, as shown in the first point of the curve (the same number of columns is achieved by Standard-NMF, but it is not lossless). The figure shows the curve representing the number of columns for different values of 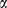, the maximum Hamming distance for which columns are merged. The curve rapidly decreases with the increase of the maximum allowed Hamming distance, as expected. Merging columns with Hamming distance at most 5 already produces a matrix with less than six thousand columns (two orders of magnitude less than the original matrix), which indicates the high amount of similar features/redundancies in the data.

**Figure 2:**
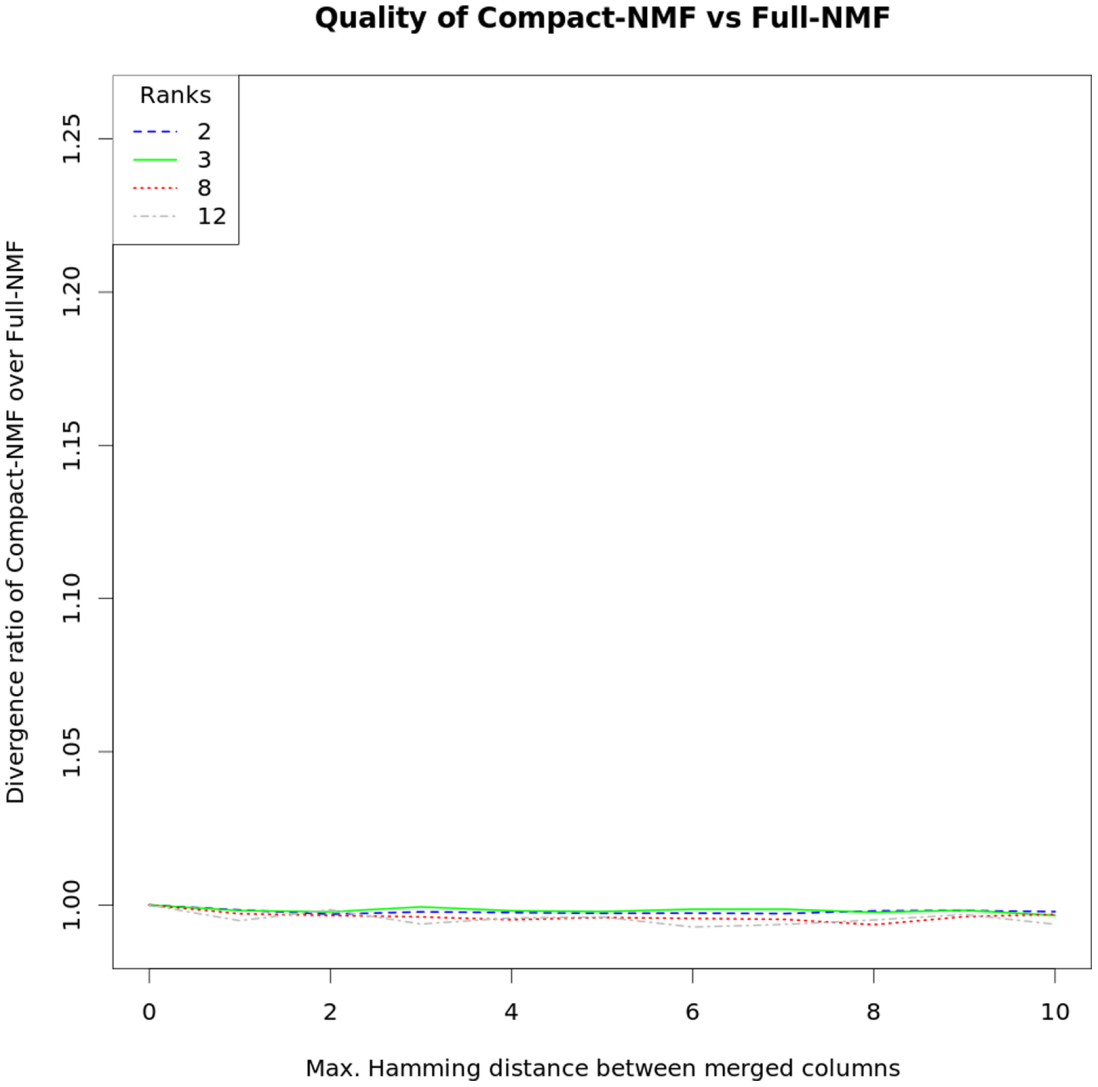
Comparison of Compact-NMF and Full-NMF over the DLBCL data of Data set 1. The matrix produced by using the DLBCL samples of Data set 1 as rows and their DNA copy number profiles as columns was used to test the distinct manners of running NMF. Full-NMF stands for the procedure which runs over all data, while Compact-NMF stands for our procedure that merge similar columns. The graphs show the ratio between the divergence (the objective function we minimize) of the Compact-NMF over the divergence of the Full-NMF, so higher values mean greater error. Results are shown for different factorization ranks.

**Figure 3:**
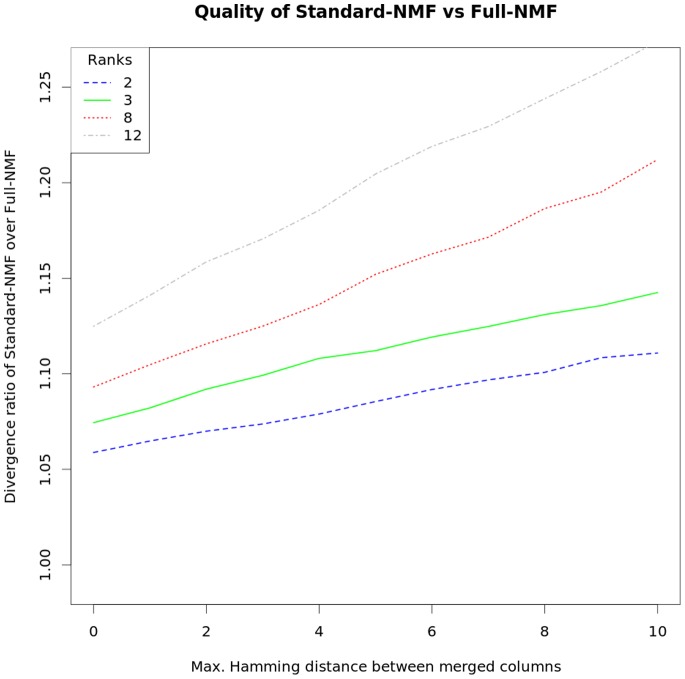
Comparison of Standard-NMF and Full-NMF over the DLBCL data of Data set 1. The matrix produced by using the DLBCL samples of Data set 1 as rows and their DNA copy number profiles as columns was used to test the distinct manners of running NMF. Full-NMF stands for the procedure which runs over all data, while Standard-NMF stands for the procedure that keeps only one of each group of similar columns. The graphs show the ratio between the divergence (the objective function we minimize) of the Standard-NMF over the divergence of the Full-NMF, so higher values mean greater error of Standard-NMF. Results are shown for different factorization ranks.

Using matrices according to these compactions, we have run Compact-NMF and Standard-NMF. Figure 2 shows the accuracy of Compact-NMF over Full-NMF, while Figure 3 shows the accuracy of Standard-NMF over Full-NMF, in terms of the achieved divergence with respect to the whole original matrix. The curves represent the ratios between the divergence achieved by the corresponding NMF and the divergence achieved by Full-NMF, on matrices generated from Data set 1 by applying the different maximum Hamming distances. To have a more accurate analysis, these ratios are in fact averaged over one hundred multiple-start runs, for each analyzed Hamming distance (variance has not been plotted because it was negligible). We see that the accuracy of Compact-NMF was essentially the same as Full-NMF for ranks between 2 and 12 and Hamming distances from 0 to 10. As a worth note, Figure 2 seems to show that Compact-NMF can even be slightly better than Full-NMF (see for instance rank 8 and Hamming distance 8, where the curve is slightly below 1). This is only possible because the internal optimization of NMF is in essence local, so the compaction of some columns kept the same quality of Full-NMF and even showed some slightly better accuracy. Such case would be obviously impossible if the internal optimization of NMF could be run to a global optimal solution (which however cannot be guaranteed).

On the other hand, Standard-NMF has much worse divergence in all cases. For example, the rank 3 curve starts with around 7% worse accuracy than Full-NMF, even for Hamming distance zero (remind that, in this case, Compact-NMF is lossless), and increases to more than 12% for Hamming distance 10. Compact-NMF and Standard-NMF have spent roughly the same amount of resources in each corresponding test case, because they basically differ in the way the matrices are built, but not in their dimensions. Finally, Full-NMF has not been run over the whole original matrix but by using Compact-NMF with Hamming distance zero (implying a lossless compaction), otherwise Full-NMF would be impractical (even with 33 thousand columns, which is the size for Compact-NMF with zero Hamming distance, it is already a quite slow and memory consuming procedure – it took around one day and 10GB of RAM in a modern desktop to perform a single factorization).

### NMF Runs and Rank Selection

As discussed in the previous section, Compact-NMF provides an accurate way to run NMF over large matrices. In order to perform cluster analysis over the four data sets, we employed the Compact-NMF method to determine the similarity matrices of patients within each data set, and subgroups were later obtained with hierarchical clustering, as described in the Section Methods. We selected Hamming distances that corresponded to approximately 1% of the information in a column, as this amount provided a good compromise of accuracy and computational time. For example, in Data Set 1, this amounts to merging probes with a maximum Hamming distance 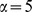. Besides that, we only grouped together probes that were not more than 500 probes distant from each other, so as to keep all the genomic profile well represented. For example, in Data set 1, the approximately 500 thousand columns were reduced to around 6 thousand columns, so as to run the experiment in a feasible time (less than half a day per factorization; total time of less than one day to run all analyses using a grid of computers) and available memory (4GB of RAM per execution). In Data set 4, the approximately 3.6 million columns were reduced to about 12 thousand columns. The total number of executions to achieve a good result was decided by analyzing the stability curve of the resulting similarity matrix with respect to the number of NMF executions, defined as the root mean-squared difference between resulting matrices of two consecutive number of executions (this was performed for each possible rank). Starting from 200 NMF executions, there was no significant difference in the resulting similarity matrix. We stopped at 300 executions, with the mean-squared difference around 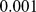 and standard deviation of 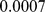. The same procedure was applied to the other data sets.

**Figure 4:**
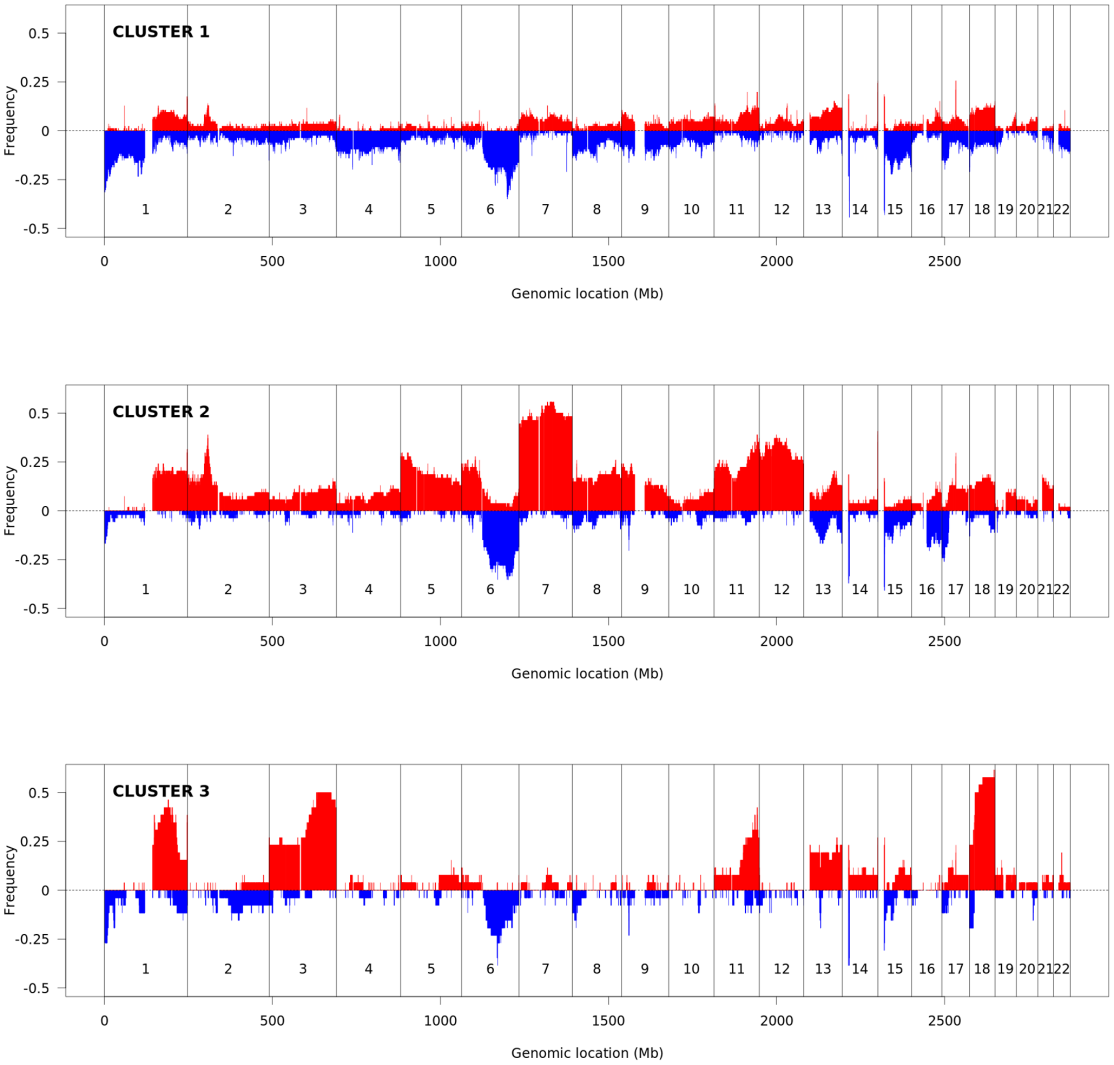
Frequency plots of CN aberrations of DLBCL patients of Data set 1, according to the clustering results. The DLBCL samples of Data set 1 were divided into three subgroups, according to the clustering results of rank 3. Clusters 1, 2 and 3 have 86, 54 and 26 cases, respectively. The frequency of samples with gain in copy number is given in red, while the frequency of losses is in blue (the scale into negative numbers is just for plotting purposes).

In order to select the appropriate clustering rank in each data set, the two measures that are detailed in the Section Methods have been used. For Data sets 1 and 4, they have selected rank 3, while for Data sets 2 and 3 they have chosen rank 2. These choices agree with most well-known quality measures in the literature (results are shown in Table S1 of the File S1) [19]–[26]. As illustration, Figure 4 shows the frequencies of CN aberrations of the three subgroups in DLBCL patients of Data set 1 (for simplicity, in the figure we considered CN 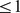 as loss and CN 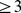 as gain). For each of the four analyzed data sets, the clustering performance measures discussed earlier have clearly indicated the rank to be used in further analyses (Table S1 in the File S1).

### Association with Molecular Subtypes and Survival Outcome

In order to show the reliability of the clustering results, we decided to evaluate their correlation with clinical and biological information of the patients. For the DLBCL data sets (Data sets 1 and 2), we considered the molecular subtypes by the cell of origin, obtained by gene expression profiling (as described in [10], [11]). Indeed, seminal gene expression profiling studies have identified at least two main subtypes of DLBCL: germinal center B-cell like (GCB) and activated B-cell like (ABC), and demonstrated that primary mediastinal B-cell lymphoma (PMBCL) is a separate disease entity [30]. Importantly, the three groups have differences in their clinical outcome and response to treatment [30]. The correlation of the clustered subgroups with cell-of-origin subtypes was tested using a Fisher exact test, according to the contingency table in Table 2. In the Data set 1, the subtype information (annotated only as ABC or GCB) was available for 57 out of 166 DLBCL cases. Even if reducing the sample size for the analysis, the smaller number of samples with subtype information does not constitute a problem, because the missingness was non-informative. We obtained p-value 0.031, indicating the existence of an association between the clustering and the cell-of-origin subtypes. From Table 2, we see that cluster 2 is particularly enriched in GCB, while cluster 3 is enriched with the ABC subtype. One can also note that cluster 1 has no predominant subtypes, which can be explained by the fact that it contains many profiles with small amount of aberrations (these samples might not even be true DLBCLs, but we prefer to refrain ourselves about such discussion, which would deviate from the goal of this paper). In the Data set 2, the data about cell-of-origin subtypes were available for 175 out of 201 patients (again, missingness is non-informative) and we obtained p-value 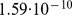 when testing the association between the two clusters and the three cell-of-origin subtypes, and p-value 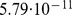 when testing the association of the two clusters with ABC vs non-ABC. In fact, we can see in Table 2 that cluster 1 is highly enriched by ABC patients. In order to better understand the division of the patients obtained with the clustering, we evaluated with the Fisher exact test the possible association between the clusters and several CN lesions known to be correlated with DLBCL cell-of-origin subtypes [12], [30]. Due to the heterogeneity of this disease, many of these aberrations are not present in the majority of the cases of any subtype. Thus, depending on the particular set of patients under examination, the subsets of lesions selected to define the clusters may differ. Regarding the DLBCL samples of Data set 1, cluster 3 (mostly enriched by ABC cases) was characterized by a significant higher frequency of gains of genes *FOXP1* (p-value = 0.009) and *BCL2* (p-value = 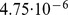), which are associated with the ABC subtype. Instead, cluster 2 (mostly enriched by GCB cases) differed from the other clusters specially in a higher presence of gains of genes *REL* (p-value = 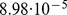), *MDM2* (p-value = 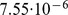) and 9p24 (p-value = 0.008), which are more associated with GCB and PMBCL subtypes. Similar patterns were observed for Data set 2. Cluster 1 (mostly enriched by ABC cases) was significantly characterized by a higher frequency of gains of genes *FOXP1* (p-value = 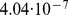), *BCL2* (p-value = 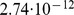) and *SPIB* (p-value = 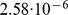), and of deletions of gene *PRDM1* (p-value = 0.003), which are all lesions correlated with the ABC subtype. Instead, cluster 2 had a significant higher proportion of gains of gene *MDM2* (p-value = 0.0006). Cluster 1 presented also a significant higher frequency (16.3% vs. 7%) of deletions of gene *PTEN* (p-value = 0.042), which is instead more associated with the GCB subtype.

**Table 2:** Association between the clusters and the molecular subtypes in the DLBCL data sets.

Cluster #	N. samples	N. samples with known subtype	ABC	GCB	PMBCL
**Data set 1**					
1	86	28	47%	53%	–
2	54	21	33.3%	66.7%	–
3	26	8	87.5%	12.5%	–
**Data set 2**					
1	86	80	68.8%	25%	6.2%
2	115	95	20%	53.7%	26.3%

This table shows the distribution of the DLBCL samples in each cluster (of rank 3 and 2, for Data set 1 and 2, respectively), with respect to the classification in molecular subtypes.

**Figure 5:**
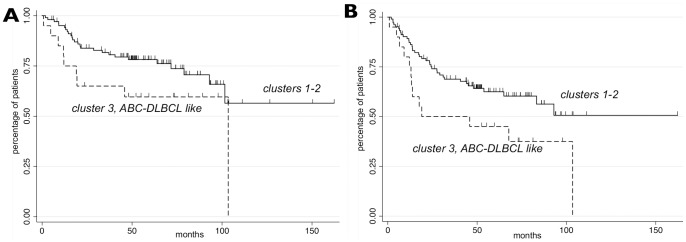
Prognostic significance of NMF-identified clusters among R-CHOP-21 treated DLBCL. Subfigures A and B show Kaplan-Meier estimates of OS (left panel, log-rank test p-value = 0.063) and PFS (right panel, log-rank test p-value = 0.034) in R-CHOP-21 treated DLBCL patients from Data set 1.

Since GCB and ABC subtypes respond differently to conventional treatments and the ABC patients have a poorer outcome [30], we also tested the association of the clustered subgroups with the survival outcome. For the evaluation, we considered the subset of Data set 1 with 124 DLBCL patients of [10], all treated with R-CHOP-21 (rituximab, cyclophosphamide, doxorubicin, vincristine and prednisone repeated every 21 days) in which both progression-free survival (that is, time to first progression or death, PFS) and overall survival (that is, time to death, OS) were available. The Kaplan-Meier estimates of the survival functions showed a similar survival behavior for clusters 1 and 2, while cluster 3, which is associated with ABC DLBCL, had a poorer outcome (for both PFS and OS). The curves are shown in Figure 5. Therefore, we applied both log-rank test and the Peto & Peto's generalization of the Wilcoxon test to compare the differences between the survival functions defined by cluster 3 and clusters 1 and 2 altogether. The tests for PFS obtained p-values 0.034 and 0.029, respectively, which suggest that patients in cluster 3 have a significant poorer PFS outcome than the others. Similar result, but not anymore significant, was achieved for OS, with p-values 0.063 and 0.051, respectively. In Data set 2, the cluster significantly presenting a profile compatible with ABC DLBCL showed a poorer overall outcome too (even if not statistically significant at level 0.05).

Of clinical relevance, the identification of cases with ABC-like and GCB-like profiles with related effects on the clinical outcome suggests that genomic DNA profiling could be evaluated in combination with immunohistochemistry, as a surrogate to predict the DLBCL subtype by cell of origin, since gene expression profiling is technically more demanding (also due to the higher stability of DNA versus RNA) and immunohistochemistry algorithms alone have not been so far fully satisfactory [31].

We also analogously analyzed the clustering results obtained with Data set 3. In this case, we tested the association between the clustering of the patients into the two groups and the status of the estrogen receptor (ER), which was available in 198 out of 200 patients. We obtained p-value 0.0375 with the Fisher exact test and, in fact, we can see in Table 3 that cluster 2 is enriched of ER-negative patients. We also tested the association between the clusters and the presence of some CN aberrations usually related to the ER-positive status [32]. Cluster 2, mostly consisting of ER-negative patients, was in fact characterized by a lower frequency of the 6q22 loss (4.8% in cluster 2 vs. 24.8% in cluster 1, with p-value = 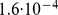). As expected, the gains of the 17q21 and 17q23 regions were equally presented in the two groups, since all patients had the amplification of gene *HER2* (situated at cytoband 17q12).

**Table 3:** Association between the clusters and the molecular subtypes in the breast cancer data set.

Cluster #	N. samples	N. samples withknown subtype	ER+	ER−
1	117	116	45%	55%
2	83	82	29%	71%

This table shows the distribution of the breast cancer patients (Data set 3) in each cluster of rank 2, with respect to the classification in molecular subtypes in estrogen receptor positive (ER+) and negative (ER−).

Finally, we have applied Compact-NMF to patients of Data set 4, whose copy number data were obtained in [14] by the Affymetrix Genome-Wide Human SNP Array 6.0 platform, thus consisting of about 1.8 million probes each. Medulloblastoma is a heterogeneous disease, and using gene expression it is possible to identify four main molecular subtypes: WNT, Sonic hedgehog (SHH), Group 3 and Group 4. This subtype information was available in 826 out of 1087 patients (non-informative missingness). The three clusters identified by Compact-NMF were significantly associated with the four subtypes (p-value 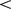 0.0001 by Fisher exact test, see Table 4). In particular, cluster 3 was highly enriched with Group 4 patients, while clusters 1 and 2 were mainly composed of SHH patients and a varying percentage of patients of the other subtypes. For this particular disease, differences between the subtypes in terms of patterns of CN aberrations are still a matter of study, and some of the few established lesions are common to more than one subtype [14], [33]. For instance, gain of chromosomes 7, 17q and 18q may characterize both Group 3 and Group 4, and the presence of these lesions has been significantly associated with our clustering subdivision (p-value 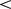 0.0001 for all of them), showing a higher frequency in cluster 3 (the one consisting mainly of these two subtypes). Also, the gain of *CDK6* is usually associated with the Group 4 subtype and is more frequent in cluster 3 (p-value 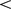 0.0001). Instead, cluster 2 is enriched of cases with gain of *GLI2*, 1q, *MYC* and loss of 5q (p-value 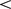 0.0001 for all of them). These aberrations are all associated with the Group 3 subtype, apart from the first of them, which has been associated with the SHH subtype.

**Table 4:** Association between the clusters and the molecular subtypes in the medulloblastoma data set.

Cluster #	N. samples	N. samples with known subtype	WNT	SHH	Group 3	Group 4
1	376	305	8.5%	49%	14%	28.5%
2	324	225	21.3%	42.2%	24.5%	12%
3	387	296	0.7%	7.1%	24%	68.2%

This table shows the distribution of the medulloblastoma patients (Data set 4) in each cluster of rank 3, with respect to the classification in the following molecular subtypes: WNT, SHH, Group 3 and Group 4.

## Conclusions

In this work we presented the Compact-NMF procedure, which specially targets the factorization of high-dimensional data sets, providing greater quality of clustering results when compared to the direct application of NMF. Using real data, we showed that Compact-NMF delivers results that are very similar to the full application of NMF over the whole data set, which cannot be done directly because of the impractical computational demand. Compact-NMF can be used to discover subgroups of patients with similar CN aberrations, which is an important task in the study of some complex and heterogeneous diseases. Our derivations were performed to work with discrete data, such as CN data, but they might extend to continuous data. For instance, if data are assumed to be generated from a Gaussian distribution, the divergence function shall be appropriately changed and Compact-NMF should be slightly modified, but the overall results would probably continue to hold.

A vast number of quality measures exist to decide the number of subgroups in an unsupervised clustering analysis. We have introduced a new quality measure to decide the appropriate number of subgroups based on the existence of robust subgroups, rather than only looking to the overall measurement obtained for each rank of the factorization (that is, for each number of subgroups). This concept is particularly suitable when we want to find biologically and/or clinically relevant subgroups that are embedded within a clustering of low quality over all subgroups, but high quality for a specific subgroup. We suggested that using many distinct quality measures that target different aspects of the clustering is the most appropriate way to choose the number of subgroups.

Four data sets of real cases were analyzed and we have obtained meaningful clustering results for all of them. In the two DLBCL data sets, the identified subgroups were associated with the cell-of-origin molecular subtypes, which have different clinical and prognostic characteristics. The subgroups identified in the breast cancer data set appeared to reflect the status of the estrogen receptor, which is a helpful biomarker for treatment decision. Finally, the subgroups found in the medulloblastoma data set were associated with the four main molecular subtypes, whose possible different targeted therapies are still under investigation. These results indicate that Compact-NMF is able to produce biological meaningful clusters in distinct scenarios.

## Supporting Information

File S1 **This file contains a detailed explanation of the reasoning behind the Compact-NMF method, including the mathematical justification for the expressions used by it.** The file also contains Table S1 with the results of well-known clustering quality measures for the data sets studied in this work.
(PDF)
