# Supplementary material for: Discovering Subgroups of Patients from DNA Copy Number Data Using NMF on Compacted Matrices
Source: PLoS One. 2013 Nov 20;8(11):e79720. doi: 10.1371/journal.pone.0079720 (PMC3835832; doi:10.1371/journal.pone.0079720)
Supplement: File S1 — This file contains a detailed explanation of the reasoning behind the Compact-NMF method, including the mathematical justification for the expressions used by it. The file also contains Table S1 with the results of well-known clustering quality measures for the data sets studied in this work. (PDF) [file pone.0079720.s001.pdf]

Supporting File S1

Discovering subgroups of patients from DNA copy number data using NMF on compacted matrices
C.P. de Campos, P.M.V. Rancoita, I. Kwee, E. Zucca, M. Zaffalon, F. Bertoni

Compact-NMF

We reduce the dimension of the matrix X by compacting the information of some of its columns (that is, SNP probes in the case of dealing with CN data) even before applying NMF. The idea is that we are given computational resources that are able to solve an NMF instance up to matrices Y of dimension $n \times p$, but we still want to approximate the (eventually much larger) $n \times m$ matrix X. The problem we want to solve is to find Y such that solving the NMF problem:

$$\operatorname*{argmin}_{W_{\hat{Y}}, H_{\hat{Y}}} \sum_{i=1}^{n} \sum_{j=1}^{p} \left(\hat{Y}(i,j) - Y(i,j) \log \hat{Y}(i,j) \right), \tag{1}$$

where $\hat{Y} = W_{\hat{Y}} \cdot H_{\hat{Y}}$, will lead to a minimum value of

$$\sum_{i=1}^{n} \sum_{j=1}^{m} \left(\hat{Y}(i, D(j)) - X(i,j) \log \hat{Y}(i, D(j)) \right), \tag{2}$$

where $D : \mathcal{N}_m \to \mathcal{N}_p$ is a partition of the m columns of X into p bins (recall that $\mathcal{N}_x$ was defined as the positive integers not greater than x).

Eq. (1) represents the NMF instance we can actually solve in a computer, while Eq. (2) is the original problem we wanted to solve (the underlying assumption is that columns from the same bin are very similar, thus estimated equally). The *Compact-NMF* directly approximates the original matrix X, still by solving the smaller NMF over Y.

Let $|D(j)| = \sum_k \mathcal{I}(D(k) = j)$ be the number of elements in the bin $j \in \mathcal{N}_p$, and apply the variable change defined by $H_{\hat{Z}}(i,j) = |D(j)| H_{\hat{Y}}(i,j)$ and $W_{\hat{Z}} = W_{\hat{Y}}$. Simple manipulations of Eq. (2) (by grouping terms of the same bin) achieve

$$\begin{aligned} &\sum_{i=1}^{n} \sum_{j'=1}^{p} \left(|D(j')| \hat{Y}(i,j') - \sum_{k=1}^{m} \mathcal{I}(D(k) = j') X(i,k) \log \hat{Y}(i,j') \right) \\ =&\sum_{i=1}^{n} \sum_{j'=1}^{p} \left(\hat{Z}(i,j') - X_D(i,j') \log \frac{\hat{Z}(i,j')}{|D(j')|} \right), \end{aligned} \tag{3}$$

where

$$X_D(i,j') = \sum_{k=1}^{m} \mathcal{I}(D(k) = j') X(i,k),$$

which in words means to sum the columns that are put together in the same bin j'. Now note that the argument $\hat{Z} = W_{\hat{Z}} \cdot H_{\hat{Z}}$ which minimizes Eq. (3) is the same as the one which minimizes

$$\operatorname*{argmin}_{W_{\hat{Z}}, H_{\hat{Z}}} \sum_{i=1}^{n} \sum_{j=1}^{p} \left(\hat{Z}(i,j) - X_D(i,j) \log \hat{Z}(i,j) \right), \tag{4}$$

because the ratio within the logarithm in Eq. (3) splits in two terms, with the second being a constant with respect to $\hat{Z}$. Hence, we can use $Y = X_D$ with the aim of (optimally) minimizing the divergence function of the NMF for X, as defined in Eq. (2). If desired, it is possible to trace back the value of $\hat{Y}$ using the relation between $\hat{Y}$ and $\hat{Z}$, but for clustering purposes this is not necessary, as $W_{\hat{Z}} = W_{\hat{Y}}$ is already the result we want.

Supplementary Tables

Table S1. Measures of rank quality for each of the four data sets.

Measure	Rank 2	3	4	5	6	7	8
**Data set 1**							
Cophenetic	**0.98**	**0.98**	0.96	0.95	0.93	0.93	0.94
Within-cluster scatter	67.5	**35.9**	59.5	82.6	99.8	107.2	112.8
Davies-Bouldin	**1.61**	1.84	1.96	3.63	1.99	2.24	2.15
Silhouette	0.58	**0.84**	0.70	0.58	0.50	0.46	0.44
Gamma statistic	0.74	**0.99**	0.96	0.94	0.84	0.88	0.79
Hubert & Levin test	0.35	**0.16**	**0.16**	0.23	0.25	0.30	0.36
AUC Elbow	0.004	0.12	**0.23**	0.21	0.21	0.23	0.24
Intra-cluster similarity	0.74	**0.84**	0.72	0.63	0.58	0.56	0.57
Maximum Intra-cluster sim.	0.76	**0.90**	0.86	0.82	0.75	0.71	0.70
**Data set 2**							
Cophenetic	**0.96**	0.87	0.90	0.90	0.92	0.92	0.92
Within-cluster scatter	**17.6**	35.5	37.6	36.5	37.8	38.7	40.3
Davies-Bouldin	1.82	3.06	**1.63**	2.35	1.92	2.29	2.51
Silhouette	**0.80**	0.50	0.53	0.56	0.54	0.52	0.49
Gamma statistic	**0.99**	0.92	0.93	0.94	0.96	0.97	0.94
Hubert & Levin test	**0.34**	0.35	0.40	0.38	0.40	0.38	0.35
AUC Elbow	0.01	0.05	0.07	0.11	0.18	0.24	**0.27**
Intra-cluster similarity	**0.82**	0.64	0.64	0.67	0.60	0.58	0.59
Maximum Intra-cluster sim.	**0.85**	0.72	0.75	0.80	0.77	0.81	0.84
**Data set 3**							
Cophenetic	**0.99**	0.95	0.95	0.93	0.92	0.93	0.93
Within-cluster scatter	**0.94**	17.2	20.7	31.4	36.4	35.5	36.3
Davies-Bouldin	**1.52**	2.09	1.80	2.19	1.87	2.03	1.87
Silhouette	**0.98**	0.75	0.75	0.55	0.50	0.53	0.55
Gamma statistic	**1.00**	0.98	0.99	0.96	0.96	0.96	0.96
Hubert & Levin test	**0.02**	0.25	0.29	0.33	0.42	0.42	0.34
AUC Elbow	**0.45**	0.36	0.34	0.37	0.39	0.41	0.44
Intra-cluster similarity	**0.98**	0.82	0.79	0.67	0.60	0.63	0.62
Maximum Intra-cluster sim.	**0.99**	0.89	0.87	0.82	0.86	0.85	0.80
**Data set 4**							
Cophenetic	0.90	**0.99**	0.96	0.96	0.94	0.94	0.93
Within-cluster scatter	117.2	**13.5**	105.2	128.8	175.3	182.3	207.0
Davies-Bouldin	**1.71**	1.99	1.98	1.84	1.93	1.82	2.26
Silhouette	0.66	**0.97**	0.74	0.69	0.57	0.57	0.48
Gamma statistic	0.91	**1.00**	0.99	0.99	0.96	0.96	0.93
Hubert & Levin test	0.35	**0.04**	0.23	0.22	0.26	0.28	0.28
AUC Elbow	0.13	0.48	0.47	0.46	0.46	0.47	**0.49**
Intra-cluster similarity	0.78	**0.97**	0.79	0.73	0.63	0.64	0.60
Maximum Intra-cluster sim.	0.81	**0.98**	0.91	0.92	0.88	0.88	0.88

Results from distinct measures do not completely agree, but overall they suggest rank 3 as the best option for Data sets 1 and 4, and rank 2 as the best option for Data sets 2 and 3.
